# Sensitive and selective determination of tetracycline in milk based on sulfur quantum dot probes

**DOI:** 10.1039/d1ra03745e

**Published:** 2021-06-29

**Authors:** Haixin Lu, Hanqiang Zhang, Yufei Li, Feng Gan

**Affiliations:** School of Chemistry, Sun Yat-Sen University Guangzhou 510275 PR China cesgf@mail.sysu.edu.cn

## Abstract

A novel fluorescent probe based on sulfur quantum dots (SQDs) was fabricated for sensitive and selective detection of tetracycline (TC) in milk samples. The blue emitting SQDs were synthesized *via* a top–down method with assistance of H_2_O_2_. The synthesized SQDs showed excellent monodispersity, water solubility and fluorescence stability, with a quantum yield (QY) of 6.30%. Furthermore, the blue fluorescence of the obtained SQDs could be effectively quenched in the presence of TC through the static quenching effect (SQE) and inner filter effect (IFE) between TC and SQDs. Under the optimum conditions, a rapid detection of TC could be accomplished within 1 min and a wide linear range could be obtained from 0.1 to 50.0 μM with a limit of detection (LOD) of 28.0 nM at a signal-to-noise ratio of 3. Finally, the SQD-based fluorescent probe was successfully applied for TC determination in milk samples with satisfactory recovery and good relative standard deviation (RSD). These results indicate that the SQD-based fluorescent probe shows great potential in practical analysis of TC in real samples with high rapidity, selectivity, and sensitivity.

## Abstract 2

A SQD-based fluorescent probe was applied to detect TC based on the inner filter effect (IFE) and static quenching effect (SQE) without any extra surface modification for the first time.

## Graphical abstract

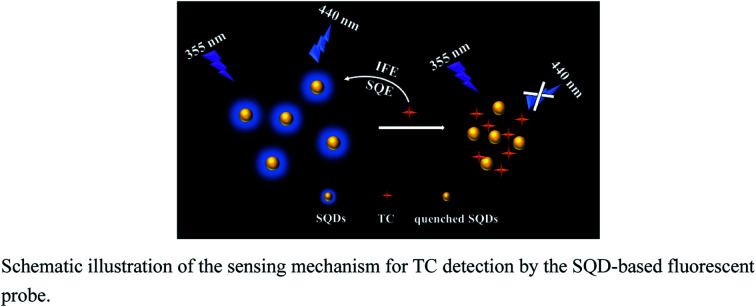

## 1. Introduction

Optical probes based on luminescent nanomaterials have drawn considerable attention due to their rapidity, sensitivity, low cost and accuracy for screening of contaminants.^1–3^ In particular, many fluorescent nanomaterials have been applied for fabrication of fluorescent probes. Zhang has reported a fluorescent probe based on lanthanum loaded graphitic carbon nitride nanosheets for Fe^3+^ detection with high selectivity and sensitivity.^4^ A fluorescent probe based on S-doped carbon dot-embedded covalent-organic frameworks (CDs@COF) was fabricated by Liu *et al.* for determination of histamine.^5^ A sensitive turn-on fluorescent probe based on MnO_2_-nanosheet-modified upconversion nanoparticles was developed by Chu *et al.* for sensitive detection of H_2_O_2_ and glucose in blood samples.^6^ However, the fabrication of fluorescent probes based on nanomaterials with high water solubility, low toxicity, and green synthesis, combined with an obvious and direct fluorescence response for target molecules still meets its limitations.

Sulfur quantum dots (SQDs), as a novel class of metal-free quantum dots, have similar advantages including good water solubility, low toxicity, and excellent biocompatibility^7–11^ compared to other metal-free quantum dots. Additionally, SQDs have been considered as a promising green fluorescent nanomaterial in recent years according to the simple and green synthesis process.^12^ SQDs were first reported in 2014 by Li's group.^11^ They applied HNO_3_ as an oxidant to slowly oxidize S^2−^ from CdS QDs to prepare SQDs. However, quantum yield (QY) of their SQDs was as low as 0.549%, and only blue light was observed. In 2018, Shen's group^13^ firstly reported a top–down method to convert sublimated sulfur into SQDs through an “assembly-fission” reaction with the QY of 3.8%. The noticeable disadvantage of Shen's method was the long synthesis time up to 125 h. In the following years, many researchers have been focusing on improving QY and shortening the reaction time including using copper-ion-assisted precipitation etching,^14^ oxygen accelerated synthesis,^15^ ultrasonication-promoted synthesis,^16^ hydrothermal reaction^17^ and ultrasonic-microwave-assisted etching methods.^18^ The SQDs reported above demonstrate particular optical properties, superior dispersibility, favorable biocompatibility, and inherent antibacterial properties, which makes them potential candidates for the fabrication of fluorescent probes. Nevertheless, the practical applications of SQD-based fluorescent probes are still in the primary stage.

Tetracycline (TC), as the most famous member of tetracyclines (TCs), is widely used for treatment of bacterial infections in humans and animals on account of its broad-spectrum antimicrobial activity, low toxicity, low cost, and good oral absorption.^19,20^ Unfortunately, the serious abuse of TC by many manufacturers due to its effectiveness and low price has exhibited several potential threats. At present, several analytical methods including microbiological,^21^ enzyme-linked aptamer assay,^22^ capillary electrophoresis,^23^ high-performance liquid chromatography^24^ and colorimetric method,^25^ have been reported for determination of TC. However, the above methods still meet limitations in long time, high cost and complex operation process, which need to be improved with advanced nanomaterials. Considering the high side effects and urgent requirement of accurate quantitative analysis of TC, it is still necessary to develop a quick and simple method for the determination of TC.^26–28^ Fabrication of fluorescent probes based on SQDs is an ideal candidate for determination of TC with high sensitivity, selectivity and accuracy.

**Scheme 1:**
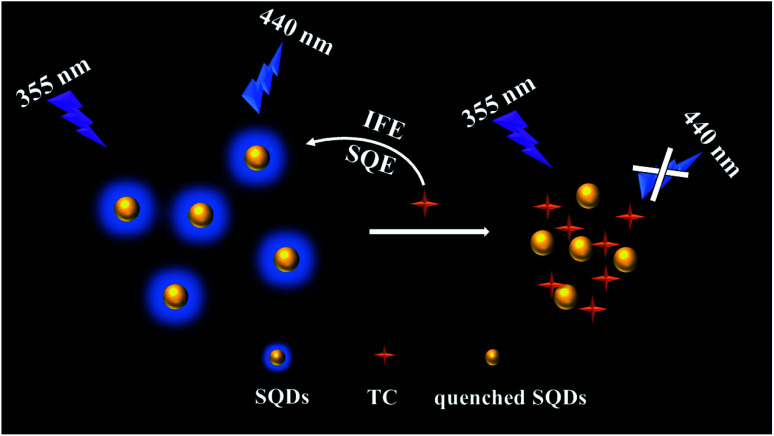
Schematic illustration of the sensing mechanism for TC detection by SQDs based fluorescent probe.

In this paper, a novel fluorescent probe based on blue emitting SQDs were fabricated for sensitive and selective detection of TC. The SQDs were synthesized *via* an H_2_O_2_-assisted top–down approach and several characterizations were conducted to verified the successful synthesis of SQDs. Furthermore, the SQDs exhibit satisfied fluorescence stability. Under this condition, the static quenching effect (SQE) and inner filter effect (IFE) and were applied to determine the content of TC based on SQDs based fluorescent probe (Scheme 1) and the linear relationship between the concentration of TC and fluorescence intensities was also investigated. Finally, the SQDs based fluorescent probe was also used to determine TC in milk samples collected from local market with satisfied results. The SQDs based fluorescent probe shows huge potentials in rapid, selective and sensitive determination of contaminates in food samples.

## 2. Experimental

### 2.1. Materials and instrumentation

Sublimed sulfur was purchased from Tianjin Fuchen Chemical Reagent Factory (Tianjin, China); Na_2_HPO_4_, NaH_2_PO_4_, NaOH, vitamin C (VC) and metal salts (Cu(NO_3_)_2_, Fe(NO_3_)_3_, Cd(NO_3_)_2_, Cr(NO_3_)_3_, Hg(NO_3_)_2_, Pb(NO_3_)_2_ Al(NO_3_)_3_, Ni(NO_3_)_2_, Co(NO_3_)_2_, Ba(NO_3_)_2_, NaCl) were acquired from Guangzhou Chemical Reagent Factory (Guangdong, China); tetracycline hydrochloride (TC), dopamine (DA), uric acid (UA), sulfamethazine (SMZ), sulfamethoxazole (SMX), quinine sulphate and glucose (Glu) were obtained from Aladdin Ltd(Shanghai, China); glutathione (GSH), cysteine (Cys), histidine (His), glycine (Gly), phenylalanine (Phe), arginine (Arg), lysine (Lys), and tyrosine (Tyr) were bought from Guangzhou Feibo Biological Technology Co., Ltd (Guangdong, China); norfloxacin (NOR) was bought from Meilun Biotechnology Co., Ltd (Dalian, China); chlortetracycline hydrochloride (CTC), oxytetracycline (OTC), amoxicillin (AMO), streptomycin (SM), gentamycin (GEN), roxithromycin (ROX) were purchased from Macklin. All reagents were analytical reagent grade and all the solutions were prepared with redistilled water.

Transmission electron microscopy (TEM) images were measured on FEI Tecnai G2 F20 (FEI Company, The Netherlands). Ultraviolet visible (UV-Vis) absorption spectra were measured by UV-2600 (SHIMADZU, Japan). Fluorescence spectra and fluorescence lifetime were measured on RF-5301 (SHIMADZU, Japan) and FLS-980 (Edinburgh, UK), respectively. Fourier transform infrared spectra (FT-IR) were measured on Thermo NICOLET AVATAR 330 (Thermo Fisher Scientific, America). X-ray photoelectron spectra was measured on Thermo Scientific Nexsa (Thermo Fisher Scientific, America). X-ray diffraction spectrum (XRD) was measured on D-MAX 2200 VPC (RIGAKU).

### 2.2. Preparation of SQDs

SQDs were prepared according to previous report with some modifications.^29^ In general, NaOH (4.0 g) and PEG-400 (3.0 mL) were dissolved in 50.0 mL of redistilled water in a round-bottom flask before gradual addition of sublimed sulfur (1.4 g). Then, the mixture was continuously stirred and heated at 70 °C for 72 h. After cooling down to room temperature, H_2_O_2_ solution (30 wt%, 2.0 mL) was quickly added into the above resulted solution with volume ratio of 2 : 5 under vigorous stirring followed by been stirred for another 30 min. Finally, the obtained yellowish solution was purified through dialysis against distilled water (500 Da, molecular weight cutoff) to obtain SQDs. The purified SQDs were stored in the refrigerator at 4 °C for further use.

### 2.3. Measurement of quantum yield

QY of SQDs was measured according to the method reported previously,^30^ using quinine sulfate (QY is 54% in 0.1 M H_2_SO_4_ solution) as standard. First, the absorbances (between 0.01 and 0.1) and fluorescent spectra of quinine sulfate and SQDs solutions were obtained at a wavelength of 355 nm. Then, the QY of SQDs was calculated by the following equation:1
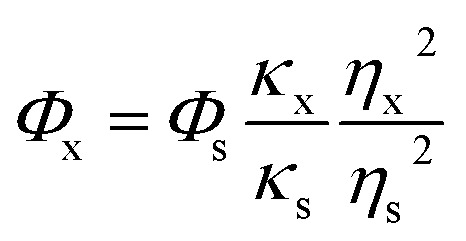
where *Φ* represents the QY. Subscript x and s represent the SQDs and quinine sulfate, respectively. *κ* is the slope obtained from the plot of the integrated fluorescence intensity *versus* absorbance, and *η* corresponds to the refractive index of solvent. *η*_x_ and *η*_s_ both are 1.33.

### 2.4. Fluorescence detection of TC

For TC determination, 2.0 mL SQDs and 250 μL phosphate buffer saline (PBS, pH 7.0, 0.2 M) were first mixed together and then added into the TC solutions with different concentrations followed by been diluted to 5.0 mL with redistilled water. The fluorescence emission spectra of the solutions were measured at an excitation wavelength of 355 nm.

### 2.5. Determination of TC in milk samples

Milk samples were obtained from local supermarket and pretreated according to previous reported method.^31^ Firstly, the proteins in the milk samples were precipitated by adding 1% (w/v) trichloroacetic acid into the samples and sonicating for 10 min. Secondly, the mixture was centrifuged at 12 000 rpm for 5 min to remove the proteins. Thirdly, the obtained supernatant was filtered through a 0.22 μm membrane to remove lipids. For standard addition recovery experiment, different concentrations including 7.0, 10.0 and 14.0 μM of TC were spiked into milk samples.

## 3. Results and discussion

### 3.1. Characterization of SQDs

**Fig. 1:**
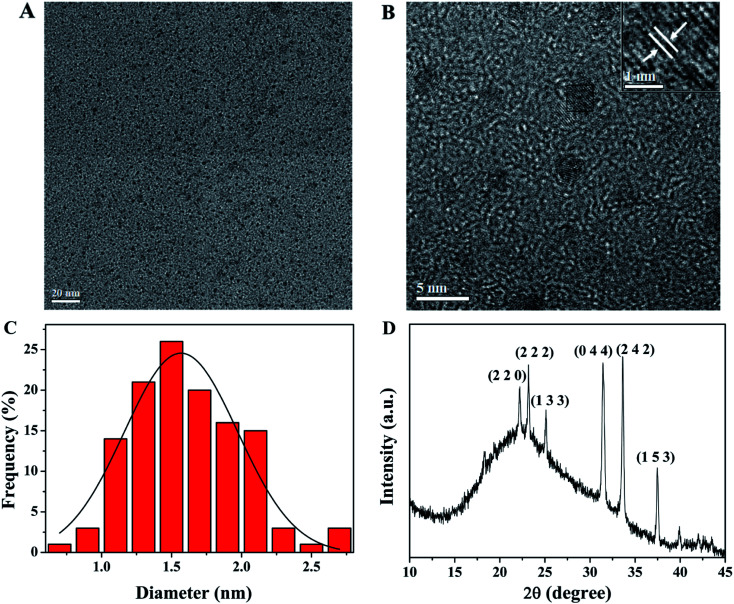
(A) TEM and (B) HR-TEM images of SQDs; (C) the size distribution of SQDs; (D) XRD pattern of SQDs.

The morphologies and size distribution of SQDs were observed by TEM. As shown in Fig. 1A, the as-prepared SQDs are well monodispersed with a nearly spherical morphology, which may be attributed to the electrostatic repulsion between the anionic groups on the surface of SQDs. HR-TEM image (Fig. 1B) presents the paralleled and ordered lattice fringes of SQDs, with a spacing of 0.20 nm. Moreover, Fig. 1C demonstrates that the size of the SQDs mainly distributes between 0.7 nm and 2.7 nm, with the average diameter of about 1.7 ± 0.4 nm (based on statistical analysis of more than 120 random SQDs in the TEM image). The XRD pattern provided in Fig. 1D shows that the positions and intensities of peaks observed at 22.2° (2 2 0), 23.2° (2 2 2), 25.1° (1 3 3), 31.4° (0 4 4), 33.6° (2 4 2) and 37.5° (1 5 3) are generally in accordance with JCPDS No. 99-0066, showing the possible formation of sulfur polycrystalline phase.^32^

**Fig. 2:**
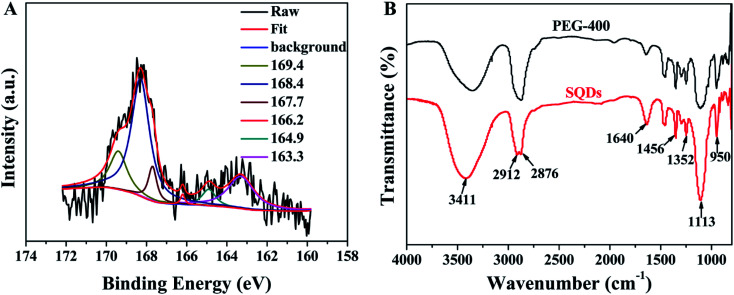
(A) High-resolution XPS spectra of S2p; (B) FT-IR spectra of PEG-400 and SQDs.

The chemical compositions and valence states of SQDs were identified by XPS. As shown in Fig. 2A, the high-resolution spectrum of S2p is deconvoluted into six peaks that indicate the existence of S[0], SO_3_^2−^ and SO_4_^2−^. The doublet located at 163.3 eV and 164.9 eV suggest the existence of S[0].^11,15^ The binding energies at 166.2 eV, 167.7 eV and 169.4 eV are attributed to SO_3_^2−^.^29^ The peak appeared at 168.4 eV comes from SO_4_^2−^.^15^ In addition, the use of passivation agent PEG-400 during the synthesis of SQDs is crucial for the fluorescence activity and stability of obtained SQDs.^13,29^ Thus, the FT-IR spectra of pure PEG-400 and the prepared SQDs were also measured. As shown in Fig. 2B, the peaks at 1456 cm^−1^, 1352 cm^−1^, 1113 cm^−1^ and 950 cm^−1^ are attributed to the existence of PEG-400 on the surface of SQDs. The peaks at 1456 cm^−1^ and 1352 cm^−1^ are both ascribed to C–H bending vibration.^33,34^ The peaks centered at 1113 cm^−1^ and 950 cm^−1^ could be attributed to the stretching vibration of C–O–H or C–O–C.^33^ The peak at 2873 cm^−1^ in PEG-400 is split into two relatively sharper peaks at 2912 cm^−1^ and 2876 cm^−1^, both of them are attributed to the stretching vibration of C–H.^35,36^ The peaks observed at 3411 cm^−1^ and 1640 cm^−1^ are belonged to –OH and C=O, respectively.^37^ No other new IR peaks are observed from the synthesized SQDs, which indicates the physical interaction between the SQDs and PEG-400 instead of chemical interaction. The XPS and FT-IR characterization results above show that the surface of the synthesized SQDs is rich in hydrophilic groups, so the obtained SQDs have high water solubility and can be used as fluorescent probes in aqueous media.

### 3.2. Optical properties of SQDs

**Fig. 3:**
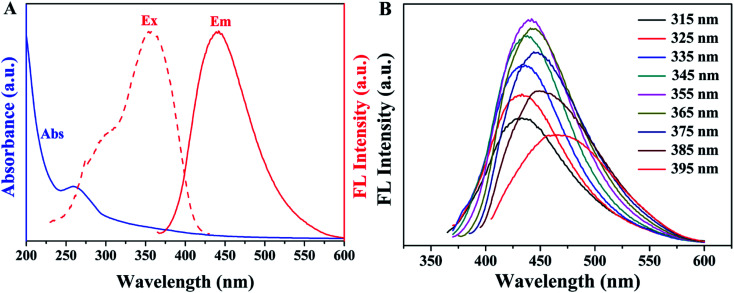
(A) UV-Vis absorption and fluorescence spectra of SQDs; (B) emission spectra of SQDs at various excitation.

To further explore optical properties of SQDs, the absorption, excitation and emission spectra of SQDs were measured. As shown in Fig. 3A, a weak peak observed at 259 nm is possibly belonged to the n–π* transition of S atoms.^38^ The fluorescence emission spectra of SQDs provided in Fig. 3B demonstrate excitation-dependent emission behavior, in which the emission intensity gradually enhances when the excitation wavelengths increase from 315 to 355 nm and then declines as excitation wavelengths further increase from 355 to 395 nm, accompanied by a red shift from 433 to 464 nm. This phenomenon, consistent with previous reports, possibly results from the inhomogeneous size distribution of particles.^29^ Meanwhile, the maximum emission peak appears at 440 nm under the excitation at 355 nm, which is the characteristic blue fluorescence of SQDs. Additionally, the QY of SQDs was calculated to be 6.30% at 355 nm excitation using quinine sulfate as the standard.

### 3.3. Fluorescence stability of SQDs

**Fig. 4:**
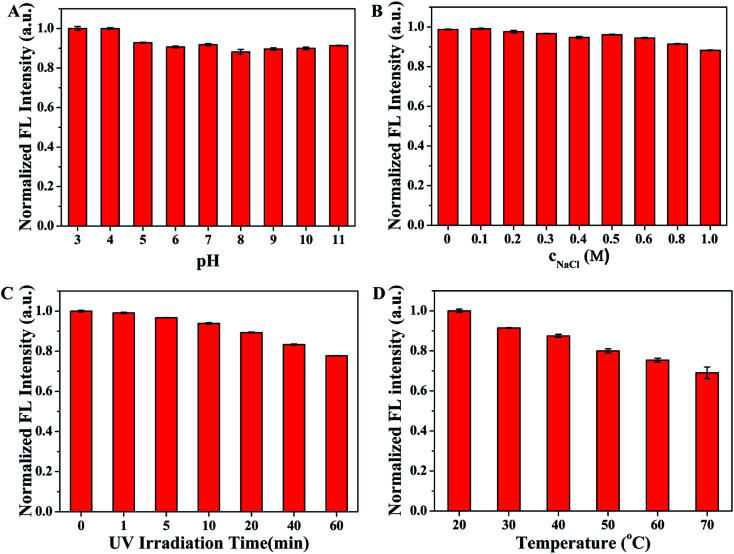
The effect of (A) pH, (B) concentrations of NaCl, (C) UV irradiation time, and (D) temperature on the fluorescence intensity of SQDs.

Fluorescence stability is of great significance to the practical sensing application of SQDs. Thus, the effects of pH value, concentrations of NaCl, UV irradiation time and temperature on the fluorescence intensity of SQDs were investigated before the further application of SQDs as fluorescent probe in sensing. As described in Fig. 4A, the normalized fluorescence intensities of SQDs remain stable and strong as the pH value varies from 3 to 11, implying the SQDs show excellent optical stability even under extreme pH conditions. Fig. 4B displays the fluorescence emission intensity at 440 nm of SQDs has negligible change when incubated with increasing NaCl concentration from 0 to 1.0 M, which suggests SQDs have good property of resisting salt effect. Moreover, continuous UV irradiation for 60 min only causes slight change in fluorescence intensity of SQDs (Fig. 4C), indicating that SQDs have good anti-photobleaching capability. In addition, it can be seen from Fig. 4D that the fluorescence intensity of SQDs is obviously affected by temperature, showing relatively poor temperature stability of SQDs. Therefore, the SQDs based fluorescent probes should be used at a constant temperature. In consideration of the accuracy of detection and the requirements of practical analysis, room temperature is chosen to carry out the subsequent experiment in this work. These results demonstrate the excellent fluorescence stability of SQDs around room temperature which guarantees the stable analytical performance.

### 3.4. Optimization of detection conditions

**Fig. 5:**
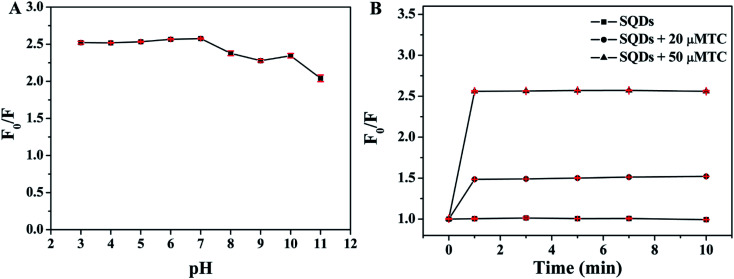
Effect of (A) pH value of PBS buffer and (B) incubation time on fluorescence quenching ratios (*F*_0_/*F*).

To achieve high sensitivity of the detection of TC, the effect of pH value of PBS buffer and incubation time on fluorescence quenching ratios (*F*_0_/*F*) were investigated, respectively (*F*_0_ and *F* represent the fluorescence intensity of SQDs in the absence and presence of TC, respectively). As depicted in Fig. 5A, obvious quenching phenomenon can be observed after addition of 50 μM TC into SQDs solution over the pH range of 3–11 and the maximum value of *F*_0_/*F* is obtained when the pH value of PBS buffer is 7.0. As such, pH 7.0 was chosen to carry out the subsequent experiment. Moreover, as illustrated in Fig. 5B, the values of *F*_0_/*F* rapidly increase in the range of 0–1 min and then remain constant after 1 min when SQDs was incubated with 20 or 50 μM TC, indicating that TC can rapidly quench the fluorescence of SQDs. Thus, 1 min was selected as the optimal incubation time.

### 3.5. Fluorescence selectivity of SQDs

**Fig. 6:**
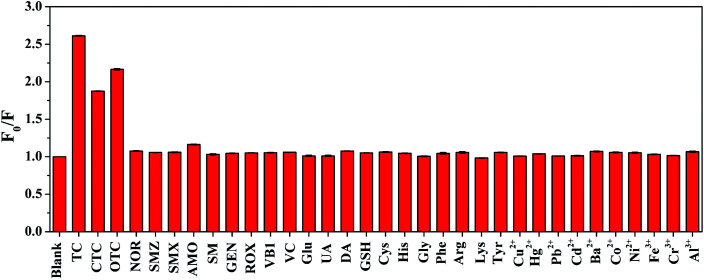
Fluorescence quenching ratios (*F*_0_/*F*) of SQDs towards different antibiotics, metal ions, and biomolecules. The concentration of TC, other antibiotics, metal ions, and VB1 are 50 μM. The concentrations of all the biomolecules except for VB1 are 500 μM.

Selectivity is a critical factor to evaluate the performance of optical sensors. To evaluate the selectivity of SQDs, the fluorescence quenching ratios towards some possible interfering species, including different antibiotics (CTC, OTC, NOR, SMZ, SMX, AMO, SM, GEN, ROX), various biomolecules (VB1, VC, Glu, UA, DA, GSH, Cys, His, Gly, Phe, Arg, Lys, Tyr) and common metal ions (Al^3+^, Ni^2+^, Co^2+^, Ba^2+^, Fe^3+^, Cr^3+^, Cu^2+^, Hg^2+^, Pb^2+^, Cd^2+^) were investigated. All the measurements were conducted under the same conditions. As shown in Fig. 6, the fluorescence quenching ratios of the nanoprobe exhibit an obvious change towards TCs (TC, CTC and OTC), while the responses towards other species are negligible. It can be concluded that the SQDs possess outstanding selectivity toward TCs, which prove the great feasibility of TCs determination based on SQDs fluorescent probe. In this study, TC with the best quenching effect and the most widely used is selected as a representative of TCs for further specific analysis.

### 3.6. Fluorescence analysis of TC

**Fig. 7:**
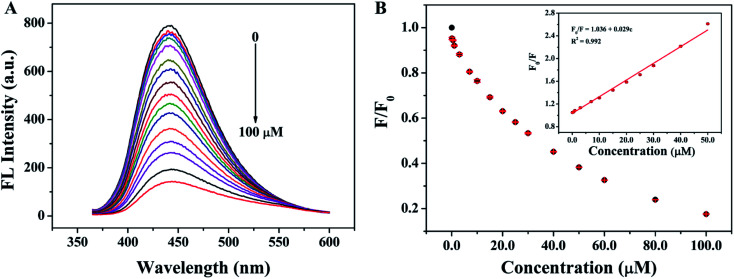
(A) Fluorescence spectra of SQDs in the presence of different concentrations of TC (0, 0.1, 0.5, 1.0, 3.0, 7.0, 10.0, 15.0, 20.0, 25.0, 30.0, 40.0, 50.0, 60.0, 80.0 and 100.0 μM); (B) the relationship between *F*/*F*_0_ and the concentrations of TC (inset: a linear relationship of *F*_0_/*F versus* the concentration of TC over the range from 0.1 to 50.0 μM).

The relationship between fluorescence intensity at 440 nm and the different concentrations of TC was evaluated by adding different concentrations of TC into the SQDs solutions and recording the fluorescence spectra under the optimal conditions. As shown in Fig. 7A, with the increasing of the concentrations of TC (from 0 to 100 μM), the fluorescence intensity of SQDs decreased significantly, indicating the good sensitivity of SQDs for TC. In addition, as shown in Fig. 7B, the fluorescence quenching ratios (*F*_0_/*F*) have a good linear relationship with the concentrations of TC (0.1–50.0 μM), and the linear regression equation is *F*_0_/*F* = 1.036 + 0.029*c* (*R*^2^ = 0.9920). The limit of detection (LOD) is 28.0 nM after calculation according to the rules of 3*σ*, which is much lower than the maximum residue limits (MRLs) of TC in milk allowed by the European Union (about 225 nM) and U.S. Food and Drug Administration (about 676 nM).^39^ Compared with other fluorescent probes reported recently (Table 1), the fluorescent probe fabricated in this work shows unique advantages in simple operation, high sensitivity, and wide linear detection range.

**Table 1:** Comparisons of different fluorescent probes for the determination of TC

Fluorescent probes	Linear range (μM)	LOD (nM)	References
IPQDs	0.5–15	76	36
Eu/CdTe QDs	0–80	2.2	39
CsPbBr_3_@BN	0.08–0.92	14	40
CDs	0.5–25	165	41
B,N-GQDs	0.04–14	1.0	42
CDs@MIPs	0.02–14	5.48	43
Pal-FL@SiO_2_-Cit-Eu	0–20	7.1	44
DPA-Ce-GMP-Eu	0.01–45	6.6	45
GQDs-Eu^3+^	0–20	8.2	46
SQDs	0.1–50.0	28.0	This work

### 3.7. Mechanism investigation

**Fig. 8:**
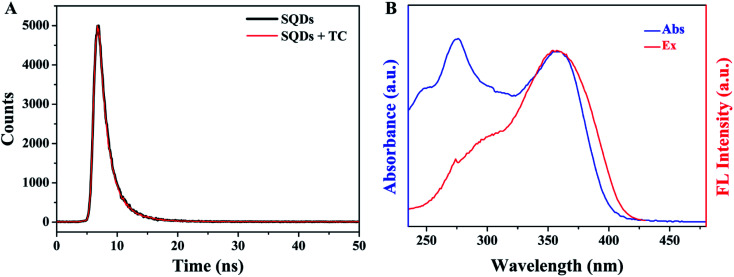
(A) The fluorescence decay curves of SQDs in the absence and presence of TC; (B) UV-Vis absorption spectrum of TC and fluorescence excitation spectrum of SQDs.

To further explore the fluorescence quenching mechanism of SQDs, some related experiments were carried out. First of all, the fluorescence lifetime of SQDs was studied and the fluorescence decay curves of SQDs in the absence and presence of TC are presented in Fig. 8A. The average luminescence lifetimes of SQDs in the absence and presence of TC are calculated to be 2.26 ns and 2.22 ns, respectively. Apparently, TC hardly affects the fluorescence lifetime of SQDs, indicating the absence of fluorescence resonance energy transfer (FRET) and dynamic quenching effect (DQE) because the fluorescence lifetime of SQDs will be shortened in the presence of TC when these two mechanisms exist.^47,48^ It can be speculated that the fluorescence quenching may result from the SQE or IFE between SQDs and TC. It is considered that the spectral overlap between absorption spectra of the quencher and the excitation spectra of the fluorophore will result in IFE.^49^ Therefore, the UV-Vis absorption spectrum of TC and excitation spectrum of SQDs were further recorded (Fig. 8B). It can be seen that the excitation spectrum of SQDs overlaps well with the UV-Vis absorption spectra of TC, which further confirms the existence of IFE. Furthermore, the Stern–Volmer equation was also utilized to describe the fluorescence quenching.^50^2
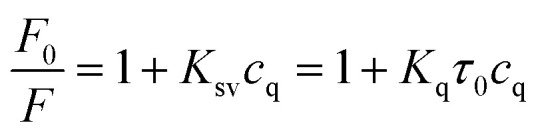
where *F*_0_ and *F* represent the fluorescence intensity of fluorophore in the absence and presence of quencher, respectively. *K*_sv_ and *c*_q_ represent quenching constant and the concentration of quencher, respectively. *K*_q_ represents the quenching rate constant and *τ*_0_ is the fluorescence lifetime of fluorophore. *K*_sv_ is approximately 2.9 × 10^4^ M^−1^ from the slope of the linear regression equation in Fig. 7B, and *K*_q_ is calculated to be 1.28 × 10^13^ M^−1^ s^−1^ based on the values of above *K*_sv_ and the fluorescence lifetime of SQDs (2.26 ns), which is significantly higher than the possible value of DQE (1.0 × 10^10^ M^−1^ s^−1^). The constant fluorescence lifetime combined with the high quenching rate constant confirm the existence of SQE.^50–52^ From the above discussion, it can be concluded that the quenching of SQDs by TC is mainly based on a combination of IFE and SQE.

### 3.8. Detection of TC in milk

In order to verify the practical applicability of SQDs based fluorescent probe in real samples analysis, the prepared SQDs were employed for the analysis of TC in milk followed by the standard addition method. As displayed in Table 2, the TC cannot be detected in milk samples and the recoveries range from 92.57% to 105.40% with low relative standard deviations (RSD) of 1.34–2.45%. These practical analysis and standard addition results indicate relative high accuracy and reproducibility for TC determination, implying the fabricated fluorescent probe is a new candidate for rapid and accurate screening of contaminate in food samples.

**Table 2:** Determination of TC in milk samples

Samples	Spiked (μM)	Measured (μM)	Recovery (%)	RSD (%)
Milk	7.0	6.48	92.57	1.72
	10.0	10.54	105.40	2.45
	14.0	14.30	102.14	1.34

## 4. Conclusion

In summary, a novel fluorescent probe based on blue emitting SQDs were fabricated for sensitive and selective detection of TC in milk samples. The synthesized SQDs showed prominent and favorable fluorescence stability. Furthermore, the fabricated fluorescent probe exhibited unique selectivity for TCs due to effective SQE and IFE between SQDs and TCs. TC was selected as a representative testing example for TCs. Under the optimum conditions, the fabricated nanoprobe exhibited a good linearity for TC from 0.1 to 50.0 μM with limit of detection (LOD) of 28.0 nM. Finally, the SQDs based fluorescent probe was also used to determine TC in milk samples with satisfied results. The SQDs based fluorescent probe shows huge potentials in rapid, selective and sensitive determination of contaminates in food samples.

## Conflicts of interest

The authors declare that they have no competing interests.

## Supplementary Material
